# Development of a Plant-Expressed Subunit Vaccine against Brucellosis

**DOI:** 10.3390/microorganisms12061047

**Published:** 2024-05-22

**Authors:** Daria A. Rutkowska, Lissinda H. Du Plessis, Essa Suleman, Martha M. O’Kennedy, Deepak B. Thimiri Govinda Raj, Yolandy Lemmer

**Affiliations:** 1 Advanced Agriculture and Food Cluster, Council for Scientific and Industrial Research, Pretoria 0001, South Africa; esuleman@csir.co.za; 2 Centre of Excellence for Pharmaceutical Sciences (PharmacenTM), North-West University, Potchefstroom 2520, South Africa; lissinda.duplessis@nwu.ac.za; 3 Future Production and Chemicals Cluster, Council for Scientific and Industrial Research, Pretoria 0001, South Africa; mokennedy@csir.co.za (M.M.O.); ylemmer@csir.co.za (Y.L.); 4 Synthetic Biology and Precision Medicine Centre, Future Production and Chemicals Cluster, Council for Scientific and Industrial Research, Pretoria 0001, South Africa; dgovindaraj@csir.co.za

**Keywords:** plant-expressed, core-like particle, CLP, new generation, vaccine, subunit, Orbivirus, *Brucella*, epitope

## Abstract

Brucellosis is an important bacterial disease of livestock and the most common zoonotic disease. The current vaccines are effective but unsafe, as they result in animal abortions and are pathogenic to humans. Virus-like particles are being investigated as molecular scaffolds for foreign antigen presentation to the immune system. Here, we sought to develop a new-generation vaccine by presenting selected *Brucella melitensis* T cell epitopes on the surface of Orbivirus core-like particles (CLPs) and transiently expressing these chimeric particles in *Nicotiana benthamiana* plants. We successfully demonstrated the assembly of five chimeric CLPs in *N. benthamiana* plants, with each CLP presenting a different T cell epitope. The safety and protective efficacy of three of the highest-yielding CLPs was investigated in a mouse model of brucellosis. All three plant-expressed chimeric CLPs were safe when inoculated into BALB/c mice at specific antigen doses. However, only one chimeric CLP induced protection against the virulent *Brucella* strain challenge equivalent to the protection induced by the commercial Rev1 vaccine. Here, we have successfully shown the assembly, safety and protective efficacy of plant-expressed chimeric CLPs presenting *B. melitensis* T cell epitopes. This is the first step in the development of a safe and efficacious subunit vaccine against brucellosis.

## 1. Introduction

Brucellosis is a contagious bacterial disease of livestock caused by facultative intracellular pathogens of the genus *Brucella* [1]. The genus *Brucella* includes at least 10 species, with *Brucella melitensis* primarily infecting sheep and goats and *Brucella abortus* primarily affecting cattle. The disease in livestock is characterised by late-term abortions, decreased fertility and reduced milk production [2], resulting in severe economic losses. Brucellosis is also the most common zoonotic disease worldwide [3], with the highly contagious *B. melitensis* considered the major causative agent of human brucellosis [4]. *Brucella* has a worldwide distribution, with different species varying in their geographic distribution [5]. *Brucella* currently affects the entire African continent, with its prevalence unknown, and is endemic to North Africa, with a heavy burden on public health, food safety and food security [6].

The current live-attenuated *Brucella* vaccines administered to animals are effective but unsafe, as they cause abortions in a proportion of pregnant animals and are also pathogenic to humans [7]. To secure the livelihoods and health of human populations affected by brucellosis, especially abattoir workers constantly at risk due to close contact with diseased animals, it is imperative that safe and efficacious alternatives to the current live *Brucella* vaccines be developed.

Virus-like particles (VLPs) are inherently immunogenic, safe, and DIVA-compliant [8]. “DIVA” is the abbreviation for Differentiating Infected from Vaccinated Animals. VLPs, formed by the assembly of viral structural proteins, exhibit a size and morphology very similar to that of wild-type virions. Proteins presented in their native conformation in repetitive arrays on the surface of VLPs stimulate humoral immunity, whilst the particulate nature of VLPs results in a broad range of T cell immune responses; hence, VLPs have been used as vaccine candidates for a range of human and animal diseases [9]. VLPs may also function as molecular scaffolds for the presentation of foreign antigens to the immune system [10,11]. Viruses from 14 different families have been used to produce chimeric VLP particles by inserting small antigenic peptides into viral structural proteins [12,13,14]. These include Orbiviruses such as bluetongue virus (BTV) and African horse sickness (AHSV), where foreign peptides were inserted into sites within the VP7 core protein and presented to the immune system on either core-like particles (CLPs) [15,16] or soluble VP7 trimers [17].

Eliciting a Th1 cellular immune response is considered key in obtaining protection against brucellosis [18]. T cell epitopes have been identified as immunogenic and protective against *B. abortus* infection in a mouse model [19]. Protection was mediated through the release of IFN-γ cytokines. Four MHC class I epitopes and one MHC class II epitope were thus selected in this study to induce a cellular T cell immune response. The sequences of these epitopes are identical to those in the *Brucella melitensis* strain and, thus, would most likely also protect sheep and goats from *B. melitensis* infection. Vaccine candidate antigens that are conserved between *B. abortus*, *B. melitensis* and *B. suis* have recently been identified [20].

Since the high costs of production and a lack of scalability derailed previous attempts to develop insect-cell-produced Orbivirus CLPs as a veterinary vaccine strategy [15,16,21], in this study, we aimed to use a well-established transient plant expression system [22]. The transient expression of heterologous proteins is mediated by either plant viral vectors, *Agrobacterium tumefaciens* bacterium or a combination of the two. The genes that are introduced into the plant cell are transiently expressed because the plant is not stably transformed, with expression only occurring for a few days. However, this short time period is sufficient for large quantities of protein to accumulate, in some cases, to more than 2 g per kilogram of plant material in one week [23]. The routine transient expression host is *Nicotiana benthamiana*, a relative of tobacco that has a fast growth rate, has a defective RNA-silencing system and is amenable to infiltration [24]. Transient plant-based production platforms thus offer several advantages over conventional mammalian, avian, insect cell, yeast and prokaryotic expression systems, including high speed, high scalability, eukaryotic post-translational protein modifications and safety due to a lack of contaminating mammalian pathogens [23,24,25]. Although glycosylation patterns differ between plant and mammalian proteins [26], and despite initial safety concerns about plant-made therapeutics [27], recent Phase I and Phase III clinical trials have demonstrated the safety of plant-made virus-like particles (VLPs) in humans [28,29,30,31,32,33]. A monoclonal antibody [34] and a Newcastle disease subunit vaccine for poultry, both plant-produced recombinant proteins, first received regulatory approval in 2006 [35]. Elelyso, a mitochondrial enzyme deficiency therapy for Gaucher disease, is the only plant-produced human therapeutic currently licensed by the US Food and Drug Administration (FDA) [36]. However, a recent phase III clinical trial with a plant-produced seasonal influenza quadrivalent VLP-based vaccine (QVLP) has successfully been concluded [30], as has a phase II/III clinical trial for a plant-produced SARS-CoV-2 VLP-based vaccine [32,37]. In addition, a large number of plant-produced human and veterinary therapeutics and vaccines have been produced and are in the pipeline for commercialization [38].

Techno-economic analyses indicate that the use of whole plants reduces upstream manufacturing costs, while the downstream processing (DSP) costs are almost equivalent to those incurred by bacterial or mammalian-based expression and account for more than 80% of total costs [23]. The reduced cost of plant molecular pharming, in addition to its other benefits, has made this strategy particularly attractive as an accessible and affordable manufacturing platform for low- and middle-income countries (LMIC’s) which do not have the infrastructure to implement other more costly expression systems [39,40]. Although there are currently no large-scale facilities for plant protein production in Africa, we hope that such a platform will be established in South Africa through the Centre for Epidemic Response and Innovation (CERI) initiative [41].

In this study, we sought to insert five different *B. melitensis* T cell epitopes into a specific insertion site within the top domain of an Orbivirus VP7 protein and assess the ability of these chimeric VP7 proteins to assemble into chimeric-core-like particles (CLPs) when transiently co-expressed with a compatible VP3 protein in *N. benthamiana* plants. The safety, immunogenicity and protective efficacy of selected plant-expressed chimeric particles was assessed in BALB/c mice, a mouse model of brucellosis.

## 2. Materials and Methods

### 2.1. Constructs

Sequences of selected *Brucella melitensis* epitopes were inserted into the Orbivirus VP7 following a strategy similar to that described previously [17]. Gene sequences encoding the native Orbivirus VP3 and VP7 proteins were obtained from the publicly accessible National Center for Biotechnology Information (NCBI) databases. The sequences were codon-optimised for optimal expression in *N. benthamiana* plant cells and subsequently inserted into the pEAQ transient expression vectors [22] via directional AgeI/XhoI restriction-enzyme-based cloning. The expression vectors were made available to the CSIR under a licence agreement from Plant Bioscience Limited (PBL), Norwich, UK.

### 2.2. Agrobacterium-Mediated Infiltration of N. benthamiana Plants

The transient expression of the Orbivirus capsid proteins in *N. benthamiana* dXT/FT plants was accomplished through *Agrobacterium*-mediated infiltration of their leaves. The protocol followed has been described in detail previously [9]. Briefly, expression constructs were transformed into *Agrobacterium tumefaciens* LBA4404 bacterial cells (Invitrogen, Waltham, MA, USA) by electroporation and propagated at 28 °C. The bacterial suspensions were combined in a 1:1 ratio for CLP assembly. The combinations were subsequently diluted with MMA buffer (10 mM MES hydrate; pH 5.6, 10 mM MgCl_2_, 100 µM 3,5-Dimethoxy-4-hydroxy-acetophenone) such that the final OD_600_ was 2. The leaves of four-week-old *N. benthamiana* dXT/FT plants were syringe-infiltrated with the agrobacterial suspensions. The plants were incubated at 27 °C for 8 days. *N. benthamiana* dXT/FT seeds were acquired from Icon Genetics GmbH under a material transfer agreement.

### 2.3. Protein Extraction and Small-Scale CLP Purification

Agroinfiltrated *N. benthamiana* leaves were harvested 8 days post-infiltration (d.p.i) and extracted and the CLPs purified as previously described [9]. The plant cell lysate was extracted in 2 volumes of VLP extraction buffer (50 mM bicine, pH = 8.4, 20 mM sodium chloride (NaCl), 0.2% Protease inhibitor cocktail P2714 (Sigma Life Science, Burlington, MA, USA)) in a multipurpose juice extractor (MATSONE, Honolulu, HI, USA) and clarified via low-speed centrifugation (7000× *g*; 20 min; 10 °C). Large plant debris was removed via filtration through 2 layers of cheesecloth. Core-like particles (CLPs) were purified using density gradient centrifugation by layering five millilitres of clarified lysate onto 60–20% iodixanol (OptiPrep™ Density Gradient Medium) (Sigma-Aldrich, St. Louis, MO, USA) gradients for analysis. Following centrifugation, 500 µL fractions were harvested using a Minipuls2 peristaltic pump (Gilson, Madison, WI, USA) and iodixanol fraction 9 subjected to electrophoresis on a 12% SDS-polyacrylamide gel followed by Coomassie Brilliant Blue G250 staining for 20 min and destaining overnight. The PageRuler^TM^ Plus prestained protein ladder (Thermo Fisher Scientific, Waltham, MA, USA) was used as a size marker. The gels were documented using a ChemiDoc™ MP Imager (Bio-Rad, Hercules, CA, USA).

### 2.4. Protein Confirmation Using LC-MS/MS-Based Peptide Sequencing

Candidate protein bands of approximately the correct size were excised from the SDS polyacrylamide gel and sent for LCMS-MS peptide sequencing analysis, previously described by our colleagues [42]. Briefly, the protein bands were in-gel trypsin-digested, resuspended in 2% acetonitrile/0.2% formic acid and analysed using a Dionex Ultimate 3000 RSLC system coupled to an AB Sciex 6600 Triple TOF mass spectrometer. The obtained MS/MS spectra were compared with the Uniprot Swissprot protein database by using Protein pilot v5, which makes use of the Paragon search engine (AB Sciex, Framingham, MA, USA). Proteins with a threshold above ≥99.9% confidence were reported.

### 2.5. Transmission Electron Microscopy (TEM)

CLPs from 35–25% iodixanol fractions were visualised by adsorbing samples onto carbon-coated holey copper grids (5 min) and stained with 2% uranyl acetate, pH = 4.2, for 15 s, as previously described [9]. Grids were air-dried and imaged using a Philips CM 10 transmission electron microscope (Philips Electron Optical Division, Eindhoven, The Netherlands) with a MegaView III side-mounted digital camera (Olympus Soft Imaging Solutions GmbH, Munster, Germany. The diameters of the particles visualised on the grid were measured using the measure tool in the iTEM Soft Imaging System software, Version 5.20 (Build 1175) (Olympus Soft Imaging Solutions GmbH, Munster, Germany). Thirty-five particles of each type were measured, and the mean diameter was calculated.

### 2.6. Antigen Preparation for Animal Trials

The antigen preparation protocol followed was similar to that of our previous animal trials [9,43]. Leaves of *N. benthamiana* dXT/FT plants were infiltrated with recombinant *Agrobacteria* combinations, and infiltrated leaves were harvested at 8 d.p.i. Following extraction, the cell extract was clarified via centrifugation (8000× *g*; 10 min; 4 °C) and filtered through a Sartoclean GF sterile midicap (3 + 0.8 µM) depth filter (Sartorius Stedim Biotech GmbH, Göttingen, Germany) and subsequently a 300 K Minimate^TM^ Tangential Flow Filtration (TFF) Capsule (Pall Life Sciences, New York, NY, USA) with the pressure not exceeding 2 Bar. Two subsequent wash steps with bicine buffer (20 mM NaCl, 50 mM bicine, pH = 8.4), each resulting in a 1:10 dilution of concentrated lysate, was performed in order to remove the protease inhibitors. The volume of the lysate was reduced to 1/10 of its original volume. The lysates were subsequently filter-sterilized through 0.45 µM + 0.2 µM Sartopore 2300 Sterile capsules (Sartorius Stedim Biotech GmbH, Göttingen, Germany) utilizing a peristaltic pump with the pressure not exceeding 2 Bar. Six-millilitre samples were analysed for protein content by density gradient centrifugation and polyacrylamide electrophoresis, as previously described, and putative CLPs were visualised via TEM. The protein content in specific fractions was quantified using a Micro BCA™ Protein Assay kit (Thermo Fisher Scientific, Waltham, MA, USA), according to the manufacturer’s instructions. The bulk of the protein in 35–25% iodixanol fractions consisted of the VP3 and VP7 proteins; however, there were some contaminating plant proteins, so the CLP quantity was slightly less than the quantities calculated. Based on the CLP quantities determined in the iodixanol fractions, we proceeded to infer the quantity of CLPs in the partially purified cell lysate.

The filter-sterilized samples were used to formulate the primary and boost inoculums for the mice in the safety and efficacy trials. These inoculums consisted of 0.4 μ, 0.8 μg or 2 μg antigen and 60% Montanide ISA 61 VG adjuvant in a total volume of 100 μL per mouse. Negative control samples were also prepared and consisted of sterile bicine buffer with 60% Montanide ISA 61 VG adjuvant in a total volume of 100 μL per mouse. The sterile samples were stored at 4 °C until use.

### 2.7. Efficacy Trials in Mice

The safety and protective efficacy of the plant-produced chimeric CLP antigens were investigated in BALB/c Mus musculus mice, a small animal model for brucellosis. Mice were bred and maintained in the small-animal facilities at the Preclinical Drug Development platform (PCDDP) unit at North-West University, Potchefstroom. All the regulatory animal ethics approvals for this study were obtained from both the CSIR and NWU animal ethics committees, and approval for the study was obtained from the Department of Agriculture, Land Reform and Rural development (DALRRD). The safety of the plant-expressed chimeric CLP antigens was investigated in fifty-five 6–8-week-old female BALB/c mice. The safety of the chimeric CLPs at 0.4 μg, 0.8 μg or 2 μg doses per animal was tested in this study, and the safety trial was conducted in three phases due to the limited numbers of animals available at any one time and for the samples to be processed efficiently. The groups, number of animals/group (n), route of administration, inoculation schedule and quantity of antigen inoculated into the animals were established from the literature [44,45,46,47]. These details for the safety trial are given in Supplementary Tables S1–S3. The mean weight of the animals prior to euthanasia was 23 g (20–28 g) (mean; range). The standard dose of the Rev1 vaccination for lambs and kids between 3 and 6 months of age is between 0.5 × 10^9^ and 2 × 10^9^ viable organisms [5]. We reduced the lowest dose by 5 logs for inoculation into mice (5 × 10^5^ viable organisms). The adjuvant selected as suitable for inoculation was Montanide ISA61VG (Seppic Speciality Ingredients Pvt. Ltd, Mumbai, India), an aqueous polymeric adjuvant for veterinary vaccines for use in livestock. It has a safety profile equivalent to aluminium salts. Mice were inoculated intraperitoneally on Day 1 and then boosted on Day 15. The mice were inspected visually 1 day post-inoculation, and thereafter, once a day to ascertain whether there were any adverse side effects caused by the inoculations, according to PCDDP SOPs. All mice, including those inoculated with the live-attenuated *Brucella* vaccine, were kept within the Biosafety Level 3 facility at the PCDDP unit during the course of this study. Mice were euthanized as per the approved protocol (cervical dislocation) on day 45. Spleens were harvested from the animals, and they underwent gross examination by the resident veterinarian. The spleens were weighed and pooled prior to processing for preliminary cellular immune response assays.

The protective efficacy of the plant-expressed chimeric CLP antigens against *Brucella melitensis* infection was investigated in fifty-five 9-week-old female BALB/c Mus musculus mice. The use of slightly older mice is advantageous, as the age at which animal models are commonly used is 8–12 week to allow for immune system development [48]. The groups, number of animals/group (n), route of administration, inoculation schedule, quantity of antigen inoculated and challenge details were obtained from the literature and are listed in Table 1. The mean weight of the animals prior to euthanasia was 24 g (19–27 g) (mean; range). The test-group mice were inoculated with either 2 μg chimeric P3 CLP antigen, 2 μg P2 CLP antigen or 2 μg of a combination of P2, P3 and P4 CLPs (0.6 μg of each CLP), with Montanide ISA61VG adjuvant. The positive-control-group mice were inoculated with the commercially available Rev1 brucellosis vaccine (Onderstepoort Biological Products (OBP)) with the titre indicated in Table 1, while the negative-control-group mice were inoculated with bicine buffer with Montanide ISA61VG adjuvant. One group of mice remained untouched. The mice were inspected visually 1 day pre-inoculation, and thereafter, once a day to ascertain whether there are any adverse side effects caused by the inoculations, according to PCDDP SOPs. The mice in the test groups, as well as those in the negative and positive control groups, were boosted with their respective antigens on day 15. On day 45, all 5 untouched mice, as well as half (5) of the mice from each of the remaining groups, were euthanized via cervical dislocation and their spleens harvested, weighed and pooled for cellular immune response assays. The remaining 5 mice from each group were challenged with the virulent *Brucella* field strain (5 × 10^5^) on day 45 of the trial. The dose of the challenge strain was as per the literature, and it was not expected that the infected BALB/c mice would display overt disease symptoms [49]. The mice were inspected twice a day for the detection of any symptoms and, in the event that the challenged mice displayed clinical symptoms of disease and/or the mice were deemed to be in pain and distress, as determined by the resident veterinarian (NWU SOP_Viv_Anim 27: Determining pain and distress in laboratory rodents), the protocol dictated that the mice would be euthanized according to NWU SOP_Viv_Anim 1: Euthanasia of rodents. No mice displayed signs of distress or pain during the course of this study and no animals were prematurely euthanized and excluded. The virulent *Brucella melitensis* strain was a field isolate obtained from the Provincial Veterinary Laboratory of the Department of Agriculture, Western Cape Government, which cultured and titrated the strain for use in the challenge experiment. It was transported to the NWU BSL-3 facility with a DALRRD red cross permit. Thirty days after challenge (Day 75), as per the protocol, the mice were euthanised via cervical dislocation and their spleens harvested for spleen bacterial cell count assays. All mice, including those inoculated with the live-attenuated *Brucella* vaccine and the virulent *Brucella* field strain, were kept within the Biosafety Level 3 facility at the PCDDP unit during the course of this study.

All animal experiments complied with the ARRIVE guidelines and were carried out in accordance with the existing and relevant national legislation and codes of practice for the Care and Use of Laboratory Animals.

Animals in each trial were randomly allocated to different cages and experimental groups. Briefly, a randomisation sequence was obtained using the Experimental Unit Randomizer software programme v1 (2004, Mr Bertus Van Zyl). Following the input of the number of experimental units (Mice), number of experiments (cages), number of experimental units per experiment (mice/cage) and number of groups per experiment (treatment groups/cage), the programme generated a randomization data sheet which indicated how to allocate mice at random to the different cages and treatment groups. The programme allocated a mouse to a different cage and treatment group simultaneously. Mice were thus randomly allocated to different cages, with each mouse in a single cage allocated randomly to a different treatment group. The mice were placed together in a large container, and the first mouse that was removed from the container was allocated to a cage and treatment according to number 1 on the randomisation data sheet. The treatment group was indicated by an ear notch in the right ear of the mouse and the cage number was indicated by an ear notch in the left ear of the mouse. This procedure was also followed until all the mice had been allocated to cages according to the numbers on the randomization data sheet.

During this study a technician (KV) was solely responsible for the antigen administration to the animals and was aware of group allocation; the researcher (DAR) performing the assays was also not blinded to group allocation and was responsible for conducting the spleen bacterial counts and performing the flow cytometry experiment. The spleen count data were analysed by another investigator (ES), while the flow cytometry data were analysed by an independent investigator (LdP). All investigators were aware of group allocation.

### 2.8. Cellular Immune Response Assays

#### 2.8.1. Spleen Homogenization

The cellular immune response assays were conducted with the cells isolated from the mouse spleens. The spleens from each mouse were aseptically harvested immediately after euthanasia; the fat surrounding this organ was removed and its weight recorded. The spleens from the mice of each group were pooled and placed in ice-cold incomplete RPMI 1640 medium (no serum, no antibiotics) in sterile 50 mL falcon tubes on ice. The spleens were homogenised through a cell strainer (BD falcon #352350) and the cells centrifuged at 300× *g* for 10 min at 4 °C. The supernatant was discarded and the cells resuspended in ice-cold ACK lysis buffer (Lonza) to lyse the red blood cells. Following a 5 min incubation step on ice, complete RPMI 1640 medium (10% FBS, no antibiotics) was added to inactivate the buffer. Following centrifugation at 300× *g* for 10 min at 4 °C, supernatant was then discarded and the cells resuspended in 5 mL RPMI medium (5% mouse serum, no antibiotics). The cells were stained with trypan blue solution, counted using a haemocytometer and diluted to 1 × 10^7^ cells/mL. An amount of 1 mL of each undiluted splenocyte suspension was added to 10 mL complete medium in 75 cm^2^ cell culture flasks and incubated O/N at 37 °C, 5% CO_2_.

#### 2.8.2. Interferon-Gamma Cytokine Secretion Assay

The detection of IFN-gamma produced from splenocytes, stimulated *in vitro* with the appropriate antigen, was performed using a MACS Mouse IFN-ɣ secretion assay detection kit (PE) (Miltenyi Biotec, Bergisch Gladbach, Germany), according to the manufacturer’s instructions. The undiluted splenocyte suspensions were seeded into 96-well flat-bottomed cell culture plates (Nunc, Roskilde, Denmark) at 0.15 × 10^7^ splenocytes/well (150 μL/well). For *in vitro* stimulation, P2, P3 and P4 peptides (Genscript, Piscataway, NJ, USA) were added to their respective wells at a final concentration of 10 μg/mL. The plate was incubated for 16 h at 37 °C, 5% CO_2_. The cytokine secretion assay was performed as per the manufacturer’s instructions. After labelling the cells with the mouse IFN-gamma detection antibody, the anti-CD4-FITC (ANTI-MO CD4 RM4-5 FITC (Invitrogen #11-0042-82)), anti-CD8-APC (ANTI-MO CD8A 53-6.7 APC Invitrogen# 17-0081-82) and B220-PerCP (PerCP anti-mouse/human CD45R/B220 BioLegend #103234) detection antibodies were also added to detect CD4+, CD8+ and B cells, respectively. The pellets were resuspended in 500 μL cold buffer (1 × PBS, 0.5% BSA, 2 mM EDTA), propidium iodide (PI, Sigma# P4864) was added to a final concentration of 0.5 μg/mL and the samples were transferred to 12 × 75 mm polystyrene test tubes for flow cytometry analysis.

#### 2.8.3. Flow Cytometry

Samples were analysed with a BD FACSVerse^TM^ flow cytometer (BD Biosciences, Franklin Lakes, NJ, USA) equipped with a 488 nm laser for the excitation of FITC, PE, PE-Cy7, PerCP-Cy5.5 and a 640 nm laser for the excitation of APC. A lymphocyte gate was used during the analysis to capture 15,000 cells. Data were analysed with FCSExpress version 7 (De Novo Software, Pasadena, CA, USA). Lymphocytes were identified on forward-scatter (FCS) and side-scatter (SSC) density plots. To ensure stringent single-cell gating, doublets were excluded using SSC and FSC height and width. Single events were gated on the FSC-H vs. FSC-W density plots, and live cells were gated with PI-negative staining. Percentages of the IFN-gamma-secreting lymphocytes as well as CD4+- and CD8+-containing lymphocytes present in the samples were quantified.

#### 2.8.4. Spleen Counts

In order to determine the protective efficacy of the plant-expressed chimeric CLPs, we needed to count the number of bacteria in the spleens of each vaccinated mouse after challenge with a virulent *Brucella* field isolate. The protocol we followed is detailed in the protocol ‘Bacterial Counts in Spleen’ [50] (www.bio-protocol.org/e954 accessed on 30 July 2018). Briefly, the spleen from each mouse was aseptically harvested immediately after euthanasia; the fat surrounding this organ removed and placed in a sterile plastic Whirl-Pak^®^ write-on bag (B01067WA (Nasco) supplied by Sigma-Aldrich, St. Louis, MO, USA) and its weight recorded. Nine parts of sterile buffer (1 × PBS, 0.1% Tween-20) was added per gram of spleen and homogenised by squeezing the organ inside the bag by hand. After homogenisation, serial dilutions of the spleen homogenates were performed (10^2^–10^5^ dilutions) with 1xPBS. An amount of 100 μL of each dilution was then dispensed in each agar plate (Tryptone Soya Agar (Thermo Fisher Scientific, Waltham, MA, USA)), and the samples were spread with a sterile Drigalski spatula until the inoculum was fully dispersed. Two agar plates were used per sample. The plates were incubated at 37° and 5% CO_2_ for 72 h. The bacterial colonies were counted on each of the plates and the spleen bacterial loads calculated by multiplying the cfus by the corresponding dilution and by 10. The average bacterial loads in the spleens, and standard deviations, were calculated using Excel. The resistance *of B. melitensis* Rev 1 and susceptibility of the *B. melitensis* challenge field strain to Streptomycin antibiotic (2.5 μg/mL) were used to distinguish between the two strains on agar plates.

## 3. Results

### 3.1. Expression and Assembly of Chimeric Orbivirus CLPs in Plants

**Figure 1:**
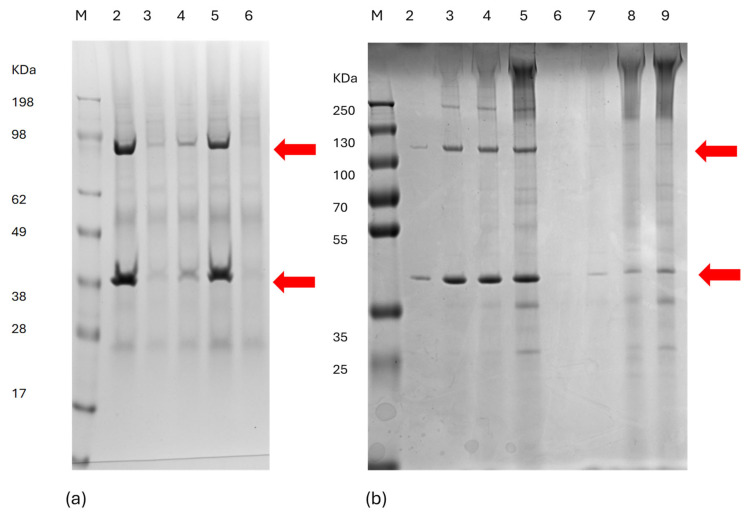
SDS-PAGE analysis of selected iodixanol fractions from gradients containing plant lysate with chimeric and wild-type (WT) Orbivirus CLPs. *N. benthamiana* dXT-FT leaves, infiltrated with recombinant Agrobacteria, were harvested 8 days post-infiltration and clarified lysates centrifuged through 60–20% iodixanol gradients. The gradients were fractionated, and 1/19 of fractions 7–10 (30–20% iodixanol) were assessed for the presence of VP7 and VP3 capsid proteins via 4–12% Bolt polyacrylamide gels. (**a**) Lane 1 contains the SeeBlue Plus 2 Prestained Protein Standard (Thermo Fisher Scientific, Waltham, MA, USA) as a marker protein (M) and the relevant sizes are indicated. Lanes 2–5 contain iodixanol fractions 9 of the WT, P1, P2 and P3 CLP gradients, respectively. Lane 6 contains an unrelated sample. (**b**) Lane 1 contains the PageRuler^TM^ Plus Prestained protein ladder (Thermo Fisher Scientific, Waltham, MA, USA) as a marker protein (M) and the relevant sizes are indicated. Lanes 2–5 contain iodixanol fractions 7–10 of the P4 CLP gradient, respectively. Lanes 6–9 contain iodixanol fractions 7–10 of the P5 CLP gradient, respectively. Red arrows indicate the position of the VP3 (103.2 KDa) and chimeric/WT VP7 (approximately 37.8 KDa) proteins on the PAGE gel.

In order to facilitate the assembly of the chimeric Orbivirus core-like particles (CLPs) in *N. benthamiana* dXT/FT plant cells, sequences encoding the structural capsid protein VP7, with or without *B. melitensis* epitope inserts (P1–P5), as well as the VP3 protein, were codon-optimised for *N. benthamiana* expression and cloned individually into the pEAQ-HT plant expression vector [22]. Leaves of 4-week-old *N. benthamiana* dXT/FT plants were infiltrated with a combination of the recombinant *Agrobacterium tumefaciens* bacteria in a ratio of 1:1 (VP7:VP3). Infiltrated leaves were harvested 8 days post-infiltration, as previously reported for optimal Orbiviral capsid protein transient expression [51], and the leaf tissue extract was centrifuged through iodixanol density gradients. The presence of the VP7 and VP3 capsid proteins within the iodixanol fractions was assessed by SDS-PAGE (Figure 1).

**Figure 2:**
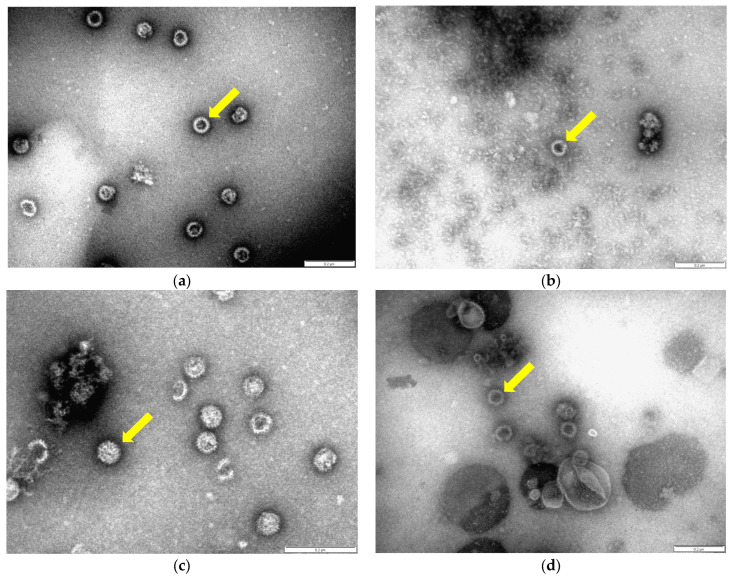

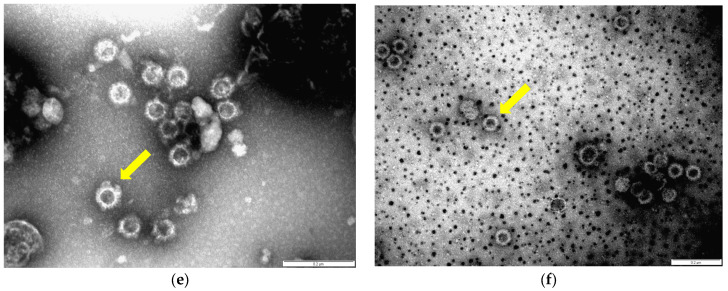
Transmission electron micrograph (TEM) images of density-gradient-purified Orbivirus CLPs containing chimeric or wild-type VP7 protein. P1 CLPs are visualised in (**a**), P2 CLPs are visualised in (**b**), P3 CLPs are visualised in (**c**), P4 CLPs are visualised in (**d**), P5 CLPs are visualised in (**e**) and wild-type (wt) CLPs are visualised in (**f**). CLPs from fraction 8 from each gradient were adsorbed onto carbon-coated holey copper grids and stained with sodium uranyl acetate. These CLPs were visualized with a Philips CM 10 transmission electron microscope. Scale bars indicate 0.2 μm. Indicated with yellow arrows are the CLPs (60 nm).

The protein bands corresponding in size to the VP3 (103.2 KDa) and chimeric/WT VP7 (approximately 37.8 KDa) capsid proteins (P1-VP7, P2-VP7 and P3-VP7) were visualised in iodixanol fraction 9 (Figure 1a, lanes 2–5). The protein bands corresponding in size to the VP3 (103.2 KDa) and chimeric VP7 (approximately 37.8 KDa) capsid proteins P4-VP7 (Figure 1b, lanes 2–5) and P5-VP7 (Figure 1b, lanes 6–9) were visualised in iodixanol fractions 7–10. The candidate P1-VP7, P2-VP7 and P3-VP7 protein bands were subjected to LC-MS/MS-based peptide sequencing analysis, and the percentage coverage values with 95% confidence of the VP7 protein were 42.7%, 36.6% and 58.4%, respectively (MS data available on request). The presence of both capsid proteins within the same gradient fractions following centrifugation indicates that these proteins assembled into high-molecular-weight particulate structures. To view these particulate structures, a sample of the 35% iodixanol gradient fraction was stained with uranyl acetate and viewed under a transmission electron microscope (TEM). The particulate structures observed (Figure 2a–f) resembled core-like particles (CLPs), as previously described [52].

These CLPs were approximately 60 nm in diameter with a ‘spiky’, knob-like surface.

Due to the low yield of the P1 CLP (Figure 1a, lane 3) and P5 CLP (Figure 1b, lanes 6–9) proteins observed on the SDS-PAGE gels, it was decided to continue the remainder of this study with the P2 (Figure 1a, lane 4), P3 (Figure 1a, lane 5) and P4 (Figure 1b, lanes 2–5) CLPs. Protein quantification of selected iodixanol fractions with the Micro BCA Protein Assay kit (Thermo Fisher Scientific, Waltham, MA, USA) indicated that the P2, P3 and P4 CLP protein yields ranged from 0.086 to 0.155 mg per gram of fresh leaf weight. The WT CLP yields were calculated to be 0.152 mg per gram fresh leaf weight.

### 3.2. Safety of Plant-Expressed Orbivirus CLPs in BALB/c Mice

The safety trial was performed with 0.4 μg, 0.8 μg and 2 μg antigen doses of the P2, P3 and P4 CLP antigens. The CLPs were partially purified by a combination of depth filtration and tangential flow filtration and subsequently quantified. The trial was performed in three phases due to the limited numbers of animals available at any one time and limited capability to process large numbers of samples. Each phase tested different concentrations of antigen, and positive and negative control groups were included in each phase. In the first phase of the trial, the test-group mice were inoculated with either 0.4 μg P2 CLP antigen or 0.4 μg wild-type CLP antigen (Table S1). The wild-type CLP antigen was included to determine whether the inserted *Brucella* epitope causes any adverse side effects. During the second phase of the trial, 0.4 μg and 2 μg antigen doses of each of the P3 and P4 CLPs were tested for their safety in mice (Table S2). During the third phase of the safety trial, 0.8 μg antigen doses of the P2, P3 and P4 CLPs were tested (Table S3). On day 45 of each phase, the mice were euthanized. No adverse effects were observed in any of the fifty-five mice during the three phases of the safety trial, and we concluded that 0.4 μg, 0.8 μg and 2 μg antigen dosages of plant-expressed chimeric P3 and P4 CLPs were safe in the mouse model of brucellosis. Due to its safety at the 0.4 μg and 0.8 μg antigen doses in mice, the 2 μg antigen dosage of the chimeric P2 CLP was also presumed to be safe in the mouse model.

### 3.3. Protective Efficacy of Plant-Expressed Orbivirus CLPs in BALB/c Mice

Next, we conducted a protective efficacy trial in fifty-five additional BALB/c mice. As we planned to challenge with a virulent *Brucella* isolate, we decided on an antigen dose of 2 μg CLP antigen/mouse. P2 and P3 CLPs were tested individually for their ability to protect mice against virulent *Brucella* challenge (2 μg/mouse), and the P2, P3 and P4 CLPs were also combined to assess whether greater protection may be elicited with a combination of CLPs (0.6 μg of each CLP). The CLPs were administered with Montanide ISA61VG adjuvant. The groups, number of animals per group, route of administration, inoculation schedule and quantity of antigen inoculated are listed in Table 1. The positive-control-group mice were inoculated with the commercially available Rev1 brucellosis vaccine (OBP), while the negative-control-group mice were inoculated with bicine buffer administered with Montanide ISA61VG adjuvant. One group of mice remained untouched. The mice in the test groups, as well as those in the negative and positive control groups, were boosted with their respective antigens on day 15. On day 45, all five untouched mice, as well as half (five) of the mice from each of the remaining groups, were euthanized and their spleens harvested for cellular immune response assays. The spleens from the mice of each group were weighed and pooled for further analysis. The remaining five mice from each group were challenged with a virulent *Brucella* field strain on day 45 of the trial. Thirty days after the challenge, as per the protocol, the mice were euthanised and their spleens harvested for spleen bacterial cell counts. Mice from both the test and control groups displayed no adverse effects during the duration of the trial, even after the challenge with the virulent Brucella strain. This result was expected from the literature, as BALB/c mice do not display symptoms of Brucella infection [49].

### 3.4. Measurement of Cellular Immune Response by Flow Cytometry and Cytokine Secretion Assay

**Figure 3:**
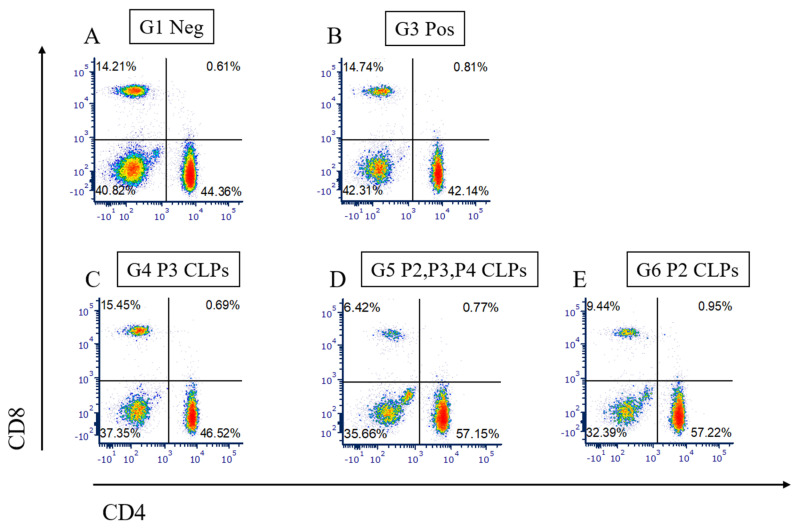
Plots of CD8+ vs. CD4+ cells in groups of animals from the animal trial. Cells from untouched animals (Group 1), Rev 1 vaccinated animals (Group 3), P3 CLP vaccinated animals (Group 4), P2, P3 and P4 vaccinated animals (Group 5) and P2 CLP vaccinated animals (Group 6) are depicted in (**A**–**E**), respectively.

Experiments performed to determine whether a cellular immune response was elicited in the mice included flow cytometry to determine the numbers of CD4+ and CD8+ cells in the antigen-stimulated splenocytes and an interferon-gamma (IFN-ɣ) cytokine secretion assay, where the secretion of IFN-ɣ from antigen-stimulated splenocytes was measured using fluorescently labelled antibodies (Figure 3).

The flow cytometry plots obtained clearly indicate an increase in the numbers of CD4+ cells (visible in the lower right-hand quadrant) in groups 5 and 6, that is, in animals inoculated with the P2, P3 and P4 CLP combination and P2 CLPs, respectively (Figure 3D,E), when compared to the numbers of CD4+ cells in the untouched (Group 1) and Rev-1 vaccinated (Group 3) animals (Figure 3A,B). The CD8+ cells (in the upper left-hand quadrant), however, decreased in groups 5 and 6. This was unexpected, as the P2 epitope is an MHC class I epitope and was intended to elicit a CD8+ cellular immune response. Group 4 (Figure 3C) had a very slight increase in both CD4+ and CD8+ cell populations when compared to Groups 1 and 3. It had been expected that the P3 epitope, being an MHC class II epitope, would elicit an increased CD4+ immune response in the mice in Group 4. Due to a lack of increase of CD4+ cells in Group 4, the increased CD4+ immune response in groups 5 and 6 was elicited not by the CLP particles themselves, but by the P2 epitope that is being presented. Interestingly, even though the P2 CLP dose in Group 5 was 1/3 of the P2 CLP dose in Group 6, the CD4+ immune response elicited was equivalent, indicating that even at lower doses (0.6 μg/mouse), P2 CLPs can elicit an equivalent immune response due to their potent immunogenicity.

**Figure 4:**
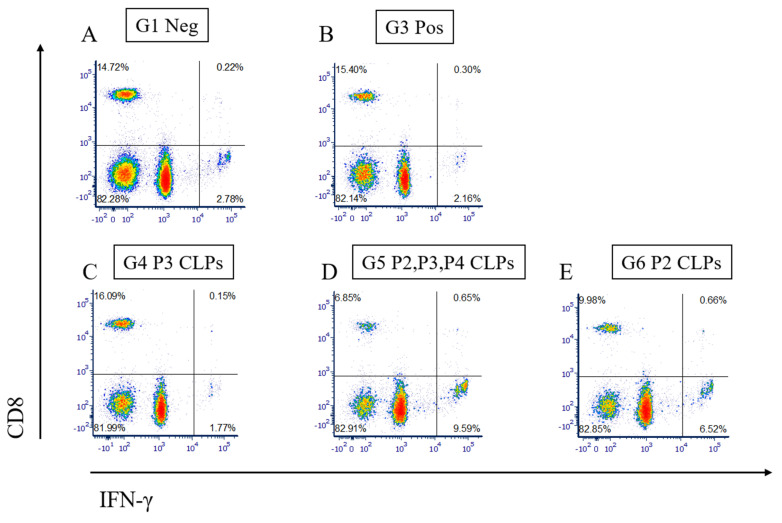
Plots of CD8+ versus IFN-gamma in groups of animals from the animal trial. CD8+ cells and IFN-gamma from untouched animals (Group 1), Rev 1 vaccinated animals (Group 3), P3 CLP vaccinated animals (Group 4), P2, P3 and P4 vaccinated animals (Group 5) and P2 CLP vaccinated animals (Group 6) are depicted in (**A**–**E**), respectively.

We also assessed whether the CD4+ and CD8+ cell populations secreted interferon-gamma (IFN-ɣ), a marker of the cellular immune response and an indication of protection against brucellosis (Figure 4).

**Figure 5:**
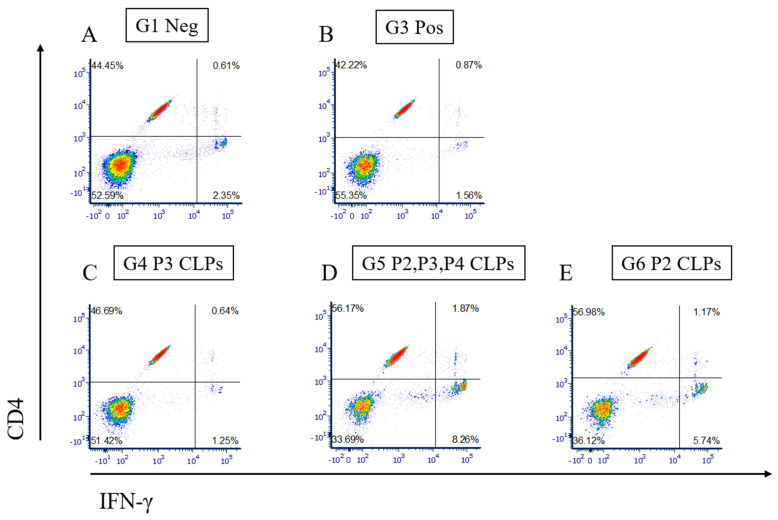
Plots of CD4+ versus IFN-gamma in groups of animals from the animal trial. CD4+ cells and IFN-gamma from untouched animals (Group 1), Rev 1 vaccinated animals (Group 3), P3 CLP vaccinated animals (Group 4), P2, P3 and P4 CLP vaccinated animals (Group 5) and P2 CLP vaccinated animals (Group 6) are depicted (**A**–**E**), respectively.

From the CD8+ versus IFN-ɣ plots obtained from the flow cytometry analysis, it is clear that there is a cell population in the lower right quadrant in groups 5 and 6 that expresses IFN-ɣ in greater quantities (Figure 4D,E) than that observed in groups 1, 3 and 4 (Figure 4A–C, respectively). This cell population is, however, clearly not composed of CD8+ cells. From the CD4+ versus IFN-ɣ plots, it also becomes apparent that a cell population observed in the lower right-hand quadrant in groups in Groups 5 and 6 (Figure 5D,E), respectively) expresses higher IFN-ɣ levels than those observed in Groups 1, 3 and 4 (Figure 5A–C, respectively). This cell population is, however, not a CD4+ population. This cell population in Groups 5 and 6 may be one of a few other cell populations known to produce IFN-ɣ. 

It must be noted at this point that the low numbers of CD4+ cells and the low levels of IFN-ɣ observed in Group 3 may be due to the experimental design and the specific peptides that were used for splenocyte stimulation. 

### 3.5. Bacterial Spleen Count Assay

**Figure 6:**
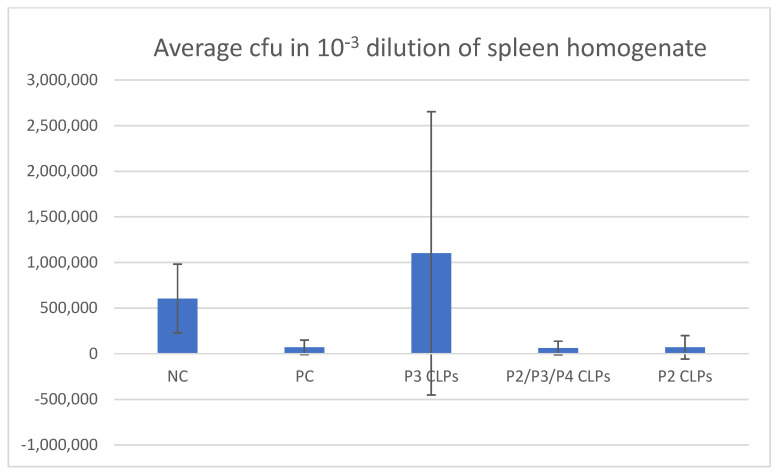
Bar graph depicting the average numbers of bacteria in the 10^−3^ dilution of the spleen homogenates of each group. The standard deviations in each group are also indicated. NC: negative control. PC: positive control.

On day 75 of the trial, spleens were harvested from the mice challenged with the virulent *Brucella* field isolate and homogenised to enable spleen bacterial count assays. A bar graph depicting the average numbers of bacteria (colony-forming units (cfu’s)) in the 10^−3^ dilution of the spleen homogenates of each group is depicted in Figure 6. The average spleen bacterial loads in groups 2–6 are listed in Table 2.

The animals in positive control Group 3 and test Group 6 inoculated with the Rev1 vaccine and P2 CLPs, respectively, yielded the same number of average colony-forming units (cfus)/spleen. Although low average cfu numbers were expected in the animals inoculated with the commercial Rev1 vaccine, the low cfu number observed in Group 6 indicates that the mice inoculated with P2 CLPS were also protected against the virulent *Brucella* strain to the same degree as the Rev1-vaccinated animals. The lowest average cfus/spleen was observed in Group 5, where the animals were inoculated with a combination of P2/P3/P4 CLPs. Although the value of 0.625 × 10^5^ average cfus/spleen of Group 5 was not much lower than that of groups 3 and 6 (0.7 × 10^5^ cfus/spleen), what is notable is that an equivalent amount of protection is observed in Group 5 and was induced by a third of the dose of each CLP (0.6 μg). The protection observed in Group 5 is most likely due to the presence of P2 CLPs. It is unlikely that P3 CLPs contributed to the protection observed in Group 5, and previous results indicate that P4 CLPs are also unlikely to contribute to the protection observed due to preliminary data obtained. Group 4 had the highest average cfus/spleen and the highest standard deviation. This is because although animals in this group exhibited differing levels of protection against *Brucella* infection, one animal (#4.9) had 8.9 times more than the average of the number of colonies in the other Group 4 animals.

## 4. Discussion

In this study, we sought to develop a plant-produced subunit vaccine against brucellosis, which is a contagious bacterial disease of livestock with a worldwide distribution and the most common zoonotic disease. This subunit vaccine would serve as a safe and effective alternative to the current live-attenuated *Brucella* vaccines, which are unsafe in animals and pathogenic towards humans [7]. The current vaccines are also not DIVA-compliant, and do not allow for the differentiation between infected and vaccinated animals.

As *Brucella* is an intracellular bacterial pathogen, the enhancement of the Th1 cellular immune response in vaccinated individuals is considered key in obtaining protection against the disease [18,53]. A previous study identified MHC-I- and MHC-II-restricted epitopes that are immunogenic and provide protection against *Brucella abortus* infection in a mouse model [19]. Protection is mediated through the release of IFN-γ cytokine. The sequences of these epitopes are identical to those in the *Brucella melitensis* strain and thus would most likely also protect sheep and goats from *Brucella* infection. In a recent study, *Brucella* antigens and polyepitopes were absorbed onto calcium phosphate nanoparticles and found to induce cross-protection against *B. melitensis* and *B. abortus* in mice [54]. Here, we proposed the presentation of epitopes on the surface of Orbivirus core-like particles (CLPs), previously employed as a particulate presentation system to induce an immune response [15,16,55]. CLPs are formed by the assembly of viral structural proteins VP3 and VP7 in repetitive arrays and can stimulate humoral immunity, whilst the particulate nature of CLPs results in a broad range of T cell immune responses. These protein-based CLPs also do not contain genomic material, which makes them safe as well as DIVA-compliant. Several positions within the top domain of Orbivirus VP7 have been identified as being good candidates for the insertion and presentation of foreign epitopes [15,16,55,56,57]. Following the investigation of possible insertion sites using protein modelling, our collaborator Dr Elien Vandermarliere (University of Gent, Belgium) identified an insertion site within the top domain of an Orbivirus VP7 for the individual insertion and presentation of four *B. melitensis* MHC class I epitopes and one MHC class II epitope [19] on the surface of CLPs. 

We subsequently co-expressed the Orbivirus VP3 and chimeric VP7 genes in *N. benthamiana* plants using the pEAQ transient plant expression system [22] and assessed CLP assembly. Novel vaccines and antibodies against animal diseases produced in plants have the advantageous of being safe, efficacious and easily scalable and requiring a relatively low capital investment [58], which makes plant biopharming an affordable and accessible manufacturing option for the developing world [39,40].

We successfully assembled all five chimeric CLPs in *N. benthamiana* plants; however, they differed in terms of yield. As very few P1 and P5 CLP proteins were visible on the SDS-PAGE gels, we proceeded to determine the yield of the P2, P3 and P4 CLPs, which ranged from 0.086 to 0.155 mg per g of fresh leaf weight, and used them for safety and efficacy trials in mice. For these trials, the CLPs were partially purified from the plant cell lysate by a combination of depth filtration and tangential flow filtration and then quantified by centrifuging a sample of the final inoculum through an iodixanol density gradient. The filtration methods used here are scalable and thus suitable for commercial manufacturing on an industrial scale. 

BALB/c mice have previously been used as a small animal model for brucellosis [19,59,60]. Although infected mice do not display overt disease symptoms [49], they do elicit a protective immune response against *Brucella* that lowers the number of pathogenic bacteria in their spleens. We first investigated the safety of the plant-produced chimeric CLPs in BALB/c mice. We decided on minimum CLP dose of 0.4 μg for this study. We also investigated 0.8 μg and 2 μg antigen doses of the chimeric CLP antigens. Wild-type CLPs were added as a control to determine whether the epitope sequences may cause adverse side effects. No adverse effects were noted in any of the mice during the three phases of the safety trial, and we concluded that the 0.4 μg, 0.8 μg and 2 μg dosages of plant-expressed chimeric P3 and P4 CLPs; 0.4 μg and 0.8 μg of the P2 CLPs; and 0.4 μg of the wild-type CLPs are safe for use in mice. Although the CLP inoculum also contained contaminating plant proteins, the safety of these plant proteins was also confirmed in this study. Indeed, these plant proteins may have an adjuvant effect resulting in an enhanced immune response against the target antigen [61] and remain to be investigated.

We proceeded with an efficacy trial to investigate whether the individual CLPs, and/or a combination of the three chimeric CLPs, could induce a protective cellular immune response against virulent *B. melitensis* challenge. As we planned to challenge with a virulent *Brucella* field isolate, we decided on an antigen dose of 2 μg CLP antigen/mouse, previously confirmed as safe in our safety trial. On day 45 of the efficacy trial, the spleens of half the number of the inoculated mice were harvested and processed for cellular immune response assays. The remaining mice were challenged with the virulent *B. melitensis* field isolate. Flow cytometry was used to determine the numbers of antigen-activated CD4+ and CD8+ cells from the processed spleens from each group as well as the identification of cells secreting IFN-ɣ, which is a key cytokine for providing protection against *Brucella* infection [62,63]. The mice in Groups 5 and 6, inoculated with a combination of P2, P3 and P4 CLPs and P2 CLPs, respectively, exhibited higher CD4+ T cell levels compared to the remaining groups. As the P3 CLPs (Group 4) and P4 CLPs (preliminary data)) did not elicit this level of CD4+ immune response, it was concluded that the P2 epitope presented on the surface of the CLPs was responsible for eliciting the CD4+ response in groups 5 and 6. The presented P2 epitope was also able to elicit an equivalent CD4+ immune response when inoculated into mice at a lower dose of 0.6 μg in combination with the other CLPs (Group 5). This result was surprising, as the P2 epitope was identified as an MCH class I epitope [19] and was expected to elicit a CD8+ cellular immune response. We also determined that a small population of cells secreting IFN-gamma in groups 5 and 6 may be either γδ T cells, natural killer (NK) cells, natural killer T cells (NKT), B cells or professional antigen-presenting cells (APCs) [64].

The average bacterial load in the spleens of the mice in Group 6, inoculated with the P2 CLPs, was equivalent to that of the average load in the spleens of the mice in Group 3 inoculated with the Rev 1 live-attenuated *B. melitensis* vaccine. This indicated that there was the same level of protection against *Brucella* infection in mice of both groups. It was expected that the Rev 1 vaccine would elicit a greater degree of protection, and thus a much lower bacterial load, in the spleens of Groups 3. This is due to the entire pathogen being able to elicit a broader range of protective responses than a single immunogenic epitope. However, it has been reported that various Rev 1 vaccines being produced around the world have differing residual virulence and immunogenicity [65], and the low protective immunogenicity being exhibited by the Rev 1 vaccine in this study may be due to the specific seed lot being propagated by the manufacturer. 

Interestingly, the average bacterial load in the spleens of the mice inoculated with a combination of P2, P3 and P4 CLPs (Group 5) was almost equivalent to that of Group 6, indicating that even at a third of the antigen dose (0.6 μg), the chimeric CLPs elicit an equivalent level of protection. The protection observed in Group 5, like in Group 6, is most likely due to the presence of P2 CLPs. The high average bacterial load observed in Group 4 in the animals inoculated with the P3 CLPs indicates that the P3 CLPs do not afford notable protection against *B. melitensis* infection and are unlikely to contribute to the protection observed in Group 5. The P4 CLPs are also unlikely to contribute to the protection observed in Group 5 due to preliminary data. 

It is of interest to note that although the increased levels of CD4+ cells and IFN-gamma cytokine in Groups 5 and 6 correlate with reduced spleen bacterial loads, these increased levels were not detected in the animals in Group 3, which were vaccinated with the Rev 1 vaccine. However, the animals in Group 3 were also protected against virulent bacterial challenge. The lack of detection of increased levels of CD4+ cells and IFN-gamma cytokine in Group 3 is likely due to the P2, P3 and P4 peptides not being presented by the Rev1 bacterium to the immune system and the harvested splenocytes thus not being stimulated when exposed to these peptides *in vitro.* This does not mean that a protective cellular immune response was not elicited in Group 3, just that perhaps the splenocytes were not stimulated *in vitro* by the specific P2, P3 and P4 peptides.

In this study, we successfully identified one *B. melitensis* epitope out of a panel of 5 protective epitope candidates that is able to elicit a protective cellular immune response in a mouse model of brucellosis. It is, however, unlikely that the protection afforded by a single epitope will afford the long-lasting protection that is required from a brucellosis vaccine. We are currently in the process of identifying additional protective *B. melitensis* epitopes that could be combined with the P2 epitope to develop a plant-produced, multi-epitope CLP-based subunit vaccine to provide robust, efficacious and long-lasting immunity against *B. melitensis*.

## 5. Conclusions

We have successfully demonstrated that the presentation of a *Brucella melitensis* T cell epitope on the surface of Orbivirus CLPs is able to elicit a protective cellular immune response in a mouse model of brucellosis. A CLP-based vaccine that affords complete protection against virulent *B. melitensis* isolates has advantages over the current Rev 1 live-attenuated *Brucella* vaccine, including safety and DIVA compliance. To develop a long-lasting protective vaccine against *B. melitensis*, we propose a multi-CLP combination vaccine containing different protective epitopes, which will subsequently be tested for immunogenicity and protective efficacy in the target animals, sheep. If successful, this will be the first plant-produced particulate subunit vaccine against brucellosis, a safe, efficacious, and DIVA-compliant alternative to the live-attenuated vaccines currently on the market.

## Figures and Tables

**Table 1:** Protective efficacy trial in 9-week-old female BALB/c mice.

Group	Primary (i.p)	Boost (i.p)	Challenge with Virulent *Brucella melitensis* Strain i.p		Animals per Group (n)
	Day 1	Day 15	Day 45	Day 75	
G1 Untouched control	5 mice untouched	5 mice untouched	5 mice sacrificed for cellular immune assays	-	5
G2 Negative control	10 mice injected with Bicine buffer + adj (100 μL/mouse)	10 mice injected with Bicine buffer + adj (100 μL/mouse)	5 mice sacrificed for cellular immune assays; 5 mice challenged with 5 × 10^5^ *B.melitensis*	5 mice sacrificed for spleen bacterial counts	10
G3 Positive control (*Brucella melitensis* vaccine Rev 1)	All 10 mice vaccinated with Rev1 5 × 10^5^	All 10 mice vaccinated with Rev1 5 × 10^5^	5 mice sacrificed for cellular immune assays; 5 mice challenged with 5 × 10^5^ *B.melitensis*	5 mice sacrificed for spleen bacterial counts	10
G4 Test Group	All 10 mice inoculated with P3 CLPs + adj (2 μg/100 μL/mouse)	All 10 mice boosted with P3 CLPs + adj (2 μg/100 μL/mouse)	5 mice sacrificed for cellular immune assays; 5 mice challenged with 5 × 10^5^ *B. melitensis*	5 mice sacrificed for spleen bacterial counts	10
G5 Test Group	All 10 mice inoculated with P2 + P3 + P4 CLPs + adj (2 μg/100 μL/mouse)	All 10 mice boosted with P2 + 3 + 4 CLPs + adj (2 μg/100 μL/mouse)	5 mice sacrificed for cellular immune assays; 5 mice challenged with 5 × 10^5^ *B. melitensis*	5 mice sacrificed for spleen bacterial counts	10
G6 Test Group	All 10 mice inoculated with P2 CLPs+ adj (2 μg/100 μL/mouse)	All 10 mice boosted with P2 CLPs + adj (2 μg/100 μL/mouse)	5 mice sacrificed for cellular immune assays; 5 mice challenged with 5 × 10^5^ *B. melitensis*	5 mice sacrificed for spleen bacterial counts	10
					55

Five mice from each group sacrificed and their spleens harvested for cellular assays (Day 45). Remaining 5 mice from each group challenged with *B. melitensis* for protection assay (Bacterial spleen counts). Five mice from each group sacrificed on Day 75 and their spleens collected for bacterial cell counts (Efficacy). I.P: Intraperitoneal; Ag: Antigen; Adj: Adjuvant; FS: Filter-sterilized.

**Table 2:** Average number of colony-forming units (cfus)/spleen in animal trial groups.

	Average cfu */Spleen
Group 2 (Negative control)	6.05 × 10^5^ (*SD* = 3.75 × 10^5^)
Group 3 (Rev1 vaccine Positive control)	0.7 × 10^5^ (*SD* = 0.8 × 10^5^)
Group 4 (P3 CLPs)	11.01 × 10^5^ (*SD* = 15.5 × 10^5^)
Group 5 (P2, P3, P4 CLPs)	0.625 × 10^5^ (*SD* = 0.75 × 10^5^)
Group 6 (P2 CLPs)	0.7 × 10^5^ (*SD* = 1.29 × 10^5^)

* cfu = colony-forming unit.

## Data Availability

The data presented in this study are available on request from the corresponding author. The commercial partner interested in this project has requested that the sensitive details of this study only be made available on request.

## Supplementary Materials

The following supporting information can be downloaded at: https://www.mdpi.com/article/10.3390/microorganisms12061047/s1, Table S1: Phase 1 of safety trial in 6–8-week-old female BALB/c mice. Table S2: Phase 2 of safety trial in 6–8-week-old female BALB/c mice. Table S3: Phase 3 of safety trial in 6–8-week-old female BALB/c mice.
