# Supplementary material for: Development of a Plant-Expressed Subunit Vaccine against Brucellosis
Source: Microorganisms. 2024 May 22;12(6):1047. doi: 10.3390/microorganisms12061047 (PMC11205566; doi:10.3390/microorganisms12061047)
Supplement: Supplementary file 1 [file microorganisms-12-01047-s001.zip › Table S2.pdf]

Table S2: Phase 2 of Safety trial in 6-8 week old female BALB/c mice

Group	Primary (i.p)	Boost (i.p)	Spleens and blood harvesting	Animal # per group
	Day 1	Day 15	Day 45	
**Untouched control**	2 mice untouched	2 mice untouched	Mice sacrificed - terminal bleed and spleens harvested for humoral and cellular immune response assays, respectively	2
**Negative control**	3 mice injected with Bicine buffer + adj (100ul/mouse)	3 mice injected with Bicine buffer + adj (100ul/mouse)	Mice sacrificed - terminal bleed and spleens harvested for humoral and cellular immune response assays, respectively	3
**Positive control (*Brucella melitensis* vaccine Rev 1)**	3 mice vaccinated with Rev1 $5x10^5$	All 3 mice vaccinated with Rev1 $5x10^5$	Mice sacrificed - terminal bleed and spleens harvested for humoral and cellular immune response assays, respectively	4
**Test Group 3**	4 mice inoculated with P4 CLPs +adj (0.4ug VLP/100ul//mouse)	4 mice boosted with P4 CLPs +adj (0.4ug VLP/100ul/mouse)	Mice sacrificed - terminal bleed and spleens harvested for humoral and cellular immune response assays, respectively	4
**Test Group 4**	4 mice inoculated with P4 CLPs + adj (2ug VLP/100ul/mouse)	4 mice boosted with P4 CLPs +adj (2ug VLP/100ul/mouse)	Mice sacrificed - terminal bleed and spleens harvested for humoral and cellular immune response assays, respectively	4
**Test Group 5**	4 mice inoculated with P3 CLPs+ adj (0.4ug VLP/100ul/mouse)	4 mice boosted with P3 CLPs +adj (0.4ug VLP/100ul/mouse)	Mice sacrificed - terminal bleed and spleens harvested for humoral and cellular immune response assays, respectively	4
**Test Group 6**	4 mice inoculated with P3 CLPs + adj (2ug VLP/100ul/mouse)	4 mice boosted with P3 CLPs +adj (2ug VLP/100ul/mouse)	Mice sacrificed - terminal bleed and spleens harvested for humoral and cellular immune response assays, respectively	4
				**25**
